# An Efficient Workflow for Screening and Stabilizing CRISPR/Cas9-Mediated Mutant Lines in *Bombyx mori*

**DOI:** 10.3390/mps4010004

**Published:** 2020-12-29

**Authors:** Daniel Brady, Alessio Saviane, Silvia Cappellozza, Federica Sandrelli

**Affiliations:** 1 Department of Biology, University of Padova, via U. Bassi 58/B, 35131 Padova, Italy; 2 Council for Agricultural Research and Economics, Research Centre for Agriculture and Environment, Sericulture Laboratory, 35143 Padova, Italy; alessio.saviane@crea.gov.it (A.S.); silvia.cappellozza@crea.gov.it (S.C.)

**Keywords:** CRISPR/Cas9, indel, molecular screening, heteroduplex assay, ARMS-PCR, *Bombyx mori*

## Abstract

The domestic silkworm *Bombyx mori* is extensively studied as a model organism for lepidopteran genetics and has an economic value in silk production. Silkworms also have applications in biomedical and cosmetic industries, and the production of mutant *B. mori* strains significantly enhances basic and applied silkworm research. In recent years, CRISPR/Cas9 technology is being rapidly adopted as the most efficient molecular tool for generating silkworm lines carrying mutations in target genes. Here we illustrate a complete and efficient workflow to screen, characterize rapidly and follow mutations through generations, allowing the generation of *B. mori* lines, stably inheriting single CRISPR/Cas9-induced mutations. This approach relies on the use of different molecular methods, the heteroduplex assay, cloning followed by Sanger sequencing, and the amplification refractory mutation system PCR. The use of these methodologies in a sequential combination allows the identification of CRISPR/Cas9-induced mutations in genes mapping on both autosomes and sex chromosomes, and the selection of appropriate individuals to found stable mutant *B. mori* lines. This protocol could be further applied to screen CRISPR/Cas9 mutations in haploid insects.

## 1. Introduction

The domesticated silkworm *Bombyx mori* has been extensively studied as a model organism, allowing the characterization of numerous biological processes [1,2], and for its economic value in silk production. In addition to the textile industry, silk fiber is also used in the biomedical and cosmetic sectors, and several studies have suggested silkworm larvae are suitable bioreactors, producing valuable recombinant proteins or modified silk useful for applied research [3]. Therefore, the production of modified or mutant *B. mori* strains has been fundamental for both basic research and applied purposes. Since Tamura and colleagues introduced the transposon-mediated germline transformation in *B. mori* [4], several techniques, including Zinc Finger Nucleases [5] and Transcription Activator-Like Effector Nucleases (TALENS) [6], have been developed to generate silkworm strains, carrying specific mutations in endogenous genes, or expressing exogenous factors.

In recent years, the Clustered Regularly Interspaced Short Palindromic Repeats (CRISPR)/Cas9 has become the dominant method of targeted mutagenesis due to its reduced costs, simplified workflow, and significantly increased efficiency [7]. The mechanism of CRISPR/Cas9 requires a guide RNA (gRNA) and Cas9 endonuclease. Briefly, the gRNA and Cas9 localize to the genomic site of interest, where Cas9 causes a double-strand break in the DNA upstream to a protospacer adjacent motif (PAM). During the subsequent DNA repair by non-homologous end-joining, random mutations frequently occur upstream of the PAM site [7]. These modifications are often indels, ranging from one to tens of base pairs (bp) that can generate null alleles, e.g., carrying premature stop codons in the coding region. Dual injections with two appropriate gRNAs have also been used to delete large fragments of DNA, spanning thousands of bp [8]. However, it has been recently shown that the inversion and reinsertion of target fragments generated by dual gRNA digestion can occur [9]. These rearrangements could generate null mutant alleles without implying a loss/acquisition of genetic material.

Since 2013, several studies on CRISPR/Cas9-mediated genome editing in *B. mori* demonstrated the possibility to generate silkworm mutants [10,11,12,13,14,15,16,17]. These studies were based on co-injection of gRNAs and Cas9 endonuclease (provided either as RNA or within expression vectors [10,11,12,13], or gRNAs and Cas9 enzyme [16]) into G_0_ embryos. Other strategies used an initial generation of independent parental silkworm lines, transgenic for constructs in which the DNA sequences for Cas9 and the selected gRNAs were cloned under the control of specific *B. mori* promoters. The CRISPR/Cas9-mediated mutagenesis was then produced in the F_1_ progeny derived from the two Cas9 and gRNA(s) parental lines [14,15,17]. The G_0_ injected adults or F1 Cas9/gRNA double transgenic progeny were mosaics for random mutations in the target genes. When these mutations occurred in germline cells, they were transmitted to subsequent generations.

Given the intrinsic nature of CRISPR/Cas9 technology, multiple types of mutations can arise independently in different germline cells giving rise to single egg broods carrying different DNA modifications in the target gene. Therefore, in order to establish silkworm lines, stably inheriting the same specific mutation, efficient and cost-effective protocols to screen, characterize, and follow DNA mutations throughout several generations are essential to select and breed suitable individuals. Several methods to screen CRISPR/Cas9 induced mutations have been developed and currently, the most commonly used in *B.mori* have been based on the PCR amplification of the target region, followed by the T7 endonuclease cleavage assay (T7EI) or by Sanger sequencing (either directly or after cloning) [2,13,17]. However, the T7EI assay is time and labor-intensive [18,19] and less sensitive compared to Sanger sequencing, which suffers from excessive costs when applied on high numbers of samples.

Here, we provide a complete and efficient workflow that allows the establishment of *B. mori* lines, stably inheriting a specific CRISPR/Cas9-induced mutation. This strategy relies on the use of different molecular protocols, including an initial heteroduplex assay to identify unknown mutations [20,21], Sanger sequencing, and the amplification refractory mutation system PCR (ARMS-PCR), used to screen for known DNA modifications [22]. The use of these protocols in a sequential combination permits the identification of CRISPR/Cas9-induced mutations, including small indels, in genes mapping on both autosomes and heteromorphic sex chromosomes, and the selection of the appropriate individuals to found a stable mutant line in *B. mori*. As an example, we use CRISPR/Cas9-mediated mutagenesis in the polyvoltine wildtype *Nistari* strain, targeting the circadian clock gene *period* (*per*), located on the sex chromosome Z, and for which ZW females are hemizygous, while ZZ males can be either hetero- or homozygous. We describe the screening process from the initial sample collection to the establishment of a *B. mori* knockout line, carrying a null allele due to a 2 bp insertion in the *per* coding region. This protocol could be further applied to identify inverted and reinserted targeted fragments induced by dual gRNA CRISPR mutagenesis.

## 2. Experimental Design

**Figure 1:**
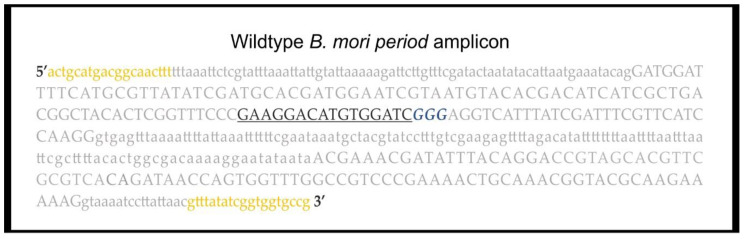
The targeted *B. mori per* region for mutagenesis. Lower- and upper-case letters identify introns and exons, respectively. Primers amplifying the 485 bp *per* fragment are in yellow. Underlined black signifies the gRNA, the PAM site is indicated with blue italics.

**Figure 2:**
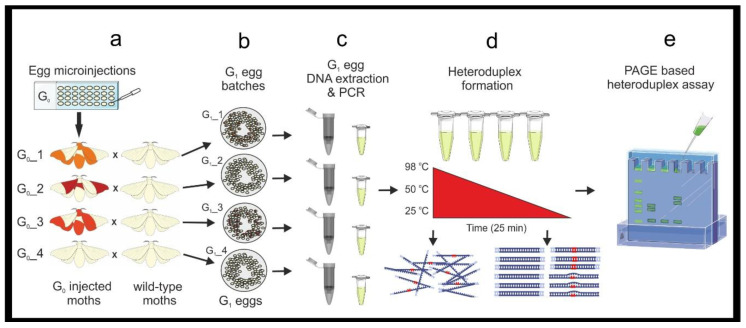
A workflow for the initial identification of mutations. (**a**) G_0_ injected eggs are reared following microinjection and crossed with wildtype moths to generate (**b**) G_1_ egg batches. (**c**) DNA extraction and PCR are performed on eggs from G_1_ egg batches followed by (**d**) temperature denaturing and heteroduplex formation. (**e**) A PAGE-based heteroduplex assay is performed to identify which egg batches contain mutations. In this scheme G_0__1 and G_0__3 moths carry mutations in germline cells and give rise to mutant G_1_ progeny. G_0__2 moth carries somatic mutations which are not transmitted to the G_1_ progeny. G_0__4 lacks any mutation.

This protocol focuses on the screening of G_1_ *B. mori* larvae following CRISPR/Cas9 mutagenesis of G_0_ injected founding individuals. To briefly overview the steps to reach this stage; an appropriate region of the target gene is identified, and suitable primers for PCR amplification are designed. In this study, we selected a 485 bp segment corresponding to the eighth and ninth exons of *per* (Figure 1). PCR amplification and direct Sanger sequencing are used on wildtype samples to ensure that the selected fragment is monomorphic among individuals of the *B. mori* strain chosen for CRISPR/Cas9-mediated mutagenesis. An appropriate single gRNA is then designed within exons contained in the target region (Figure 1). Next, the PCR amplicon is used to confirm that the gRNA and Cas9 efficiently localize to and digest the DNA, in vitro. Once gRNA efficacy has been confirmed, microinjections into fertile *B. mori* eggs are performed to generate the G_0_ injected moths (Figure 2).

Molecular screening for the establishment of a stable homozygous gene knockout line involves three primary steps; (1) initial mutant screening with heteroduplex assay, (2) mutation sequence determination with TA cloning and Sanger sequencing, and (3) specific mutation screening by ARMS-PCR. Some authors begin initial screening on G_0_ injected larvae; however, these animals may be mosaics without the potential for germline transmission of the observed DNA modification. Furthermore, tissue extraction from *B. mori* larvae is an invasive procedure that can result in mortality. Therefore, we rear all G_0_ larvae without interference and begin molecular screening on G_1_ egg batches.

### 2.1. Initial Identification of Mutations

The initial G_1_ embryo screening for mutations (Figure 2) involves a heteroduplex assay, which allows for the identification of the egg batch/es carrying promising mutations and is based on PCR amplification and PAGE. There is an option here on the approach that can be taken and should be determined by the number of putative mutated G_1_ egg batches that are being analyzed (see Notes 1 and 2, Section 3.1.1).

### 2.2. Mutation Sequence Determination

**Figure 3:**
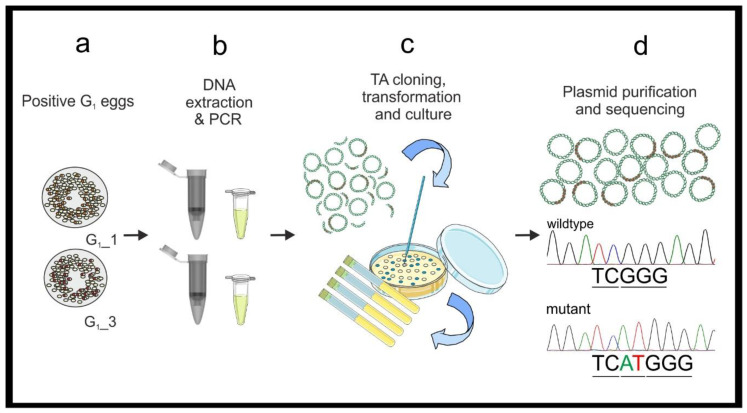
A workflow for mutation determination. (**a**,**b**) DNA extraction and PCR are performed on individual eggs from egg batches bearing a mutation. (**c**) The PCR fragments are cloned in plasmids for sequencing. (**d**) Plasmids are purified, and specific DNA sequences are determined with Sanger sequencing. The precise mutation sequence is used to design ARMS-PCR primers.

The molecular characterization of the mutations is performed using TA cloning and Sanger sequencing on G_1_ embryo DNA (Figure 3). This step allows for the determination of the actual nature of the mutation/s and the designing of suitable primers required for the subsequent ARMS-PCR screening step (Section 2.3 and Section 3.2). Molecular characterization requires PCR amplification, cloning into a TA cloning vector followed by Sanger sequencing and basic computational analyses, then designing appropriate primers to amplify a 400 to 1000 bp region around the genomic target. The primer couples employed to verify the gRNAs specificity in vitro can be used at this stage. Cloning requires both a cloning vector system and competent bacterial cells. We have used the StrataClone PCR Cloning Kit, which ligates PCR amplicons into a TA cloning vector that are transformed with kit specific competent bacteria. For sequencing, many companies provide plasmid primers with optimized running parameters. If this option is not available, it is possible to employ the PCR primers used for cloning. Subsequently, a sequence alignment must be performed to identify each specific mutation and determine whether a stop codon will be produced in the protein sequence. These computational analyses can be completed with a large number of software packages. We describe the process (Supplementary Methods A) with the use of three freely available software packages; CLC Sequence Viewer (V.8, Qiagen), MEGA 7 and Serial Cloner.

### 2.3. Hemolymph Sampling and Screening with ARMS-PCR

**Figure 4:**
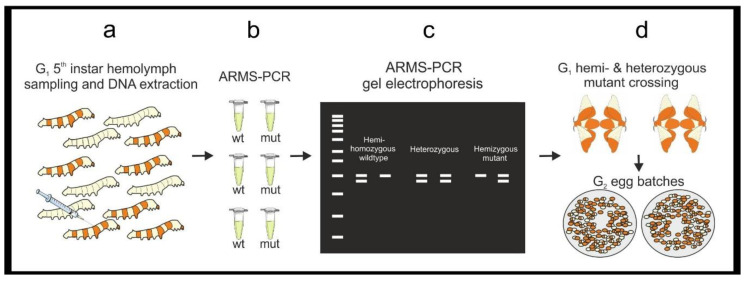
A workflow for hemolymph sampling, DNA extraction and screening with ARMS-PCR. (**a**) G_1_ larvae bearing a target mutation are reared to the 5th instar. At the beginning of the 5th instar, hemolymph is sampled and DNA is extracted to perform (**b**,**c**) ARMS-PCRs identifying individual larvae carrying the mutation. (**d**) Appropriate larvae are then reared to adults and bred to generate G_2_ egg batches bearing hemi-, hetero- and homozygote mutant individuals.

Screening for heterozygous, homozygous, and hemizygous mutants is comprised of (i) hemolymph sampling from larvae, (ii) DNA extraction, and (iii) screening by ARMS-PCR, which allows for the identification of the mutation-bearing organisms (Figure 4). These are then selected for the generation of the stable mutant line.

**Figure 5:**
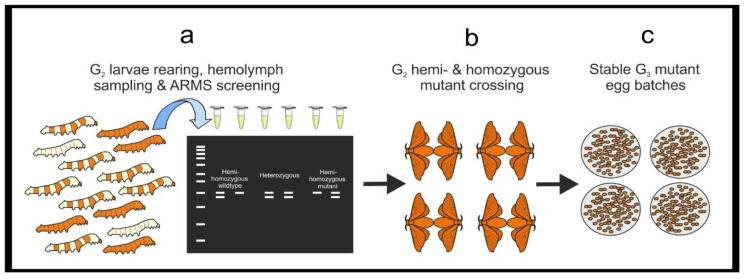
A workflow for ARMS screening of G_2_ larvae and establishment of a stable mutant line. (**a**) G_2_ larvae are reared to the 5th instar and ARMS-PCR is performed on DNA purified from their hemolymph. G_2_ hemi- and homozygous mutant larvae are reared until adult stage and (**b**) bred to generate (**c**) G_3_ lines stably inheriting the mutation.

Hemolymph sampling is a critical step since it may have deleterious effects on larval development, increasing the mortality rate. However, in trialing non-invasive DNA extraction methods using frass (feces) and exuviae (cast-off outer skins of the larvae at the molting stage) we achieved poor and inconsistent results. Here we describe our optimized protocol to extract *B. mori* hemolymph minimizing mortality. Regarding the DNA extraction procedure, we found that the PureYield Plasmid Miniprep System (Promega, Dane County, WI, USA) provides the highest quality and quantity of DNA (600 ng when eluted in 30 μL of H_2_O) from 20 μL of hemolymph. We also achieved good results using the Wizard^®^ Genomic DNA Purification Kit (Promega, Dane County, WI, USA) and the GeneJet Genomic DNA purification kit (ThermoFisher, Waltham, MA, USA), while a 10% Chelex buffer did not produce reproducible outcomes. Following sampling, G_1_ larvae must be reared individually; we find Petri dishes ideal housing as the larvae can be observed without disturbance and are easy to stack, secure, and transport. The ARMS-PCR method screens for known mutations by PCR and electrophoresis [16]. ARMS-PCR requires two PCR reactions for each sample, one containing amplification primers for the wildtype sequence and the second containing primers specifically designed to amplify the mutant haplotype (e.g., a modified forward primer bearing the mutated sequence at its 3′ region). For each reaction, it is suggested to add an internal PCR control, co-amplifying any genomic region easily distinguishable from the ARMS-PCR amplicons on an agarose gel. We amplify a 500 bp sequence of the gene *cycle* (*cyc)* as an internal control. ARMS-PCR is repeated with G_2_ animals, selecting hemi- or homozygous mutant females and males, which will establish the stable mutant line (from G_3_ onward) (Figure 5).

### 2.4. Materials

- Ice and ice box
- Masking tape
- Lab tissue
- Forceps (PharmaPlast, Thessaloniki, Greece; Cat. no.: 161)
- 15 mL sterile tubes (SARSTEDT, Nümbrecht, Germany; Cat. no.: 62.554.502)
- 50 mL sterile tubes (SARSTEDT, Nümbrecht, Germany; Cat. no.: 62.547.254)
- 1.5 mL sterile microtubes (SARSTEDT, Nümbrecht, Germany; Cat. no.: 72.706.200)
- μStripPro 0.2 mL PCR tubes (SARSTEDT, Nümbrecht, Germany; Cat. no.: 72.985.99)
- Hypodermic needle (32G) (BioTekne SRL, Bologna, Italy; Cat. no.: AM32G)
- Pipette tips (P10), (SARSTEDT, Nümbrecht, Germany; Cat. no.: 70.3010)
- Pipette tips (P200), (SARSTEDT, Nümbrecht, Germany; Cat. no.: 70.3030.020)
- Pipette tips (P1000), (SARSTEDT, Nümbrecht, Germany; Cat. no.: 70.3050.20)
- Sterile vented Petri dishes, 94 mm (Roll S.A.S., Sacco, Italy; Cat. no.: 182480)
- 70% ethanol (VWR, Milan, Italy; Cat. no.: 20821.310)
- TAE buffer (50×: Tris free base: 242 g, disodium EDTA 18.61 g, glacial acetic acid 57.1 mL, double-distilled H_2_O to 1 L)
- TBE buffer (5×: 104 g tris base 27.5 g, boric acid 40 mL, 0.5 M EDTA, 700 mL double-distilled H_2_O, pH to 8.0, adjust to 1 L)
- LB Broth Miller (Merck Life Science, Milano, Italy; Cat. no.: L3522)
- Phusion Green Hot Start II High Fidelity PCR Master Mix (ThermoFisher, Waltham, MA, USA, Cat. no.: F566L)
- DreamTaq Green PCR Master Mix (2X) (ThermoFisher, Waltham, MA, USA, Cat. no.: K1082)
- Agarose (EuroClone, Milano, Italy; Cat. no.: EMR920500)
- Ethidium bromide (Merck Life Science, Milano, Italy; Cat. no.: E1510)
- 1 Kb Plus DNA Ladder (ThermoFisher, Waltham, MA, USA, Cat. no.: 10787018)
- Acrylamide/Bis-acrylamide 30% (29:1) (Merck, Darmstadt, Germany; Cat. no.: A3574)
- Ammonium persulphate (APS; Merck Life Science, Milano, Italy; Cat. no.: A3678)
- N, N, N′, N′-Tetramethylethylenediamine (TEMED; Merck Life Science, Milano, Italy; Cat. no.: T9281)
- StrataClone PCR Cloning Kit (Agilent Technologies, Santa Clara, CA, USA; Cat. no.: 240205)
- Ampicillin (Merck Life Science, Milano, Italy; Cat. no.: 59349).

### 2.5. Equipment

- Micropipettes (P10, P200, P1000)
- 1.5 mL Microtube racks
- Gel Doc™ XR+ (Bio-Rad, Hercules, CA, USA; Cat. no.: 1708182)
- NanoDrop 2000c (Thermo Scientific, Waltham, MA, USA; Cat. no.: ND-2000C)
- Thermal Cycler Veriti™ (Thermo Scientific, Waltham, MA, USA; Cat. no.: 4375786)
- Vert Slab (Amersham Bioscience, San Francisco, CA, USA).

## 3. Procedure

### 3.1. Initial Identification of Mutations

#### 3.1.1. Genomic DNA Extraction from G_1_ Embryos, and PCR. Time for Completion: DNA Extraction-90 min. Per 24 Egg Samples. PCR and Agarose Gel Electrophoresis-4 h (Figure 2)

1. Following CRISPR/Cas9 microinjection, rear the G_0_ individuals until adult stage as in [23,24] and outbreed singly with wildtype moths from the same genetic background. After egg-laying, label each egg batch with a unique code.
2. Allow the G_1_ eggs to develop for three days, collect five eggs per egg batches, place each egg into a microtube. Label microtubes with the unique egg batch code, and replicate number (see Note 1).
3. Crush the eggs with a pipette tip and perform DNA extraction and purification using the chosen DNA purification kit following the manufacturer’s instructions. Quantify DNA with a NanoDrop and prepare 20 ng/μL working solutions for each sample.
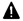
**CRITICAL STEP** Also perform DNA extraction on wildtype tissue from the same genetic background. This DNA will be used to obtain amplicons required for the mixed heteroduplex assay.
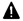
**CRITICAL STEP** Use high fidelity polymerase when performing initial molecular screening. Calculate a sufficient PCR master mix volume for all your samples in 20 μL PCR reactions, include negative and positive controls. Calculate the required volume of wildtype amplicon (for heteroduplex formation, a minimum of 10 μL of wildtype amplicon per sample: e.g., 10 μL × 12 samples = 120 μL).
6. Prepare a PCR master mix (see primer sequences in Figure 1 and Table 1), for 480 μL add reagents as in Table 2. Label and add 19 μL of PCR master mix to each PCR tube. Add 1 μL of the 20 ng/μL genomic DNA (gDNA) samples to each tube. Add 20 ng of wildtype gDNA to the positive control and wildtype PCR reactions, and 1 μL of H_2_O to the negative control. Briefly vortex and centrifuge. Run the PCR; 98 °C–2 min, [98 °C–20 s, 62 °C–30 s, 72 °C 20 s] × 30, 72 °C 2–min and hold at 4 °C.
7. Load 5 μL of the PCR reactions on a 1.2% agarose gel (TAE buffer) stained with ethidium bromide. Run the gel for 30 min. at 100 V and image on a GelDoc XR+, to confirm all the PCR reactions have been successful.

#### 3.1.2. Heteroduplex Assay and Polyacrylamide Gel Electrophoresis. Time for Completion: Preparation for Heteroduplex Formation-90 min. PAGE Gel Preparation–3 h; Preparing, Loading and Running the PAGE Gel–4 h

1. Prepare and label a PCR tube for each egg sample as well as positive and negative controls. To each tube, add 5 μL of the egg sample PCR amplicon.
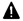
**CRITICAL STEP** Add 5 μL of wildtype PCR amplicon to each PCR sample, vortex and centrifuge the mixtures. Place the samples in a thermocycler and perform a temperature denaturing ramp-down; 98 °C for 3 min. and decrease the temperature 1 °C every 20 s. Hold the ramp-down at 25 °C. If mutations are present in a G_1_ sample, heteroduplexes will have formed. Store the samples at 4 °C until use or at −20 °C for longer storage. Keep in mind that samples from hemizygous females bearing a *per* mutation will not produce heteroduplexes without the addition of the wildtype amplicon.
3. Under a chemical hood prepare a 15% non-denaturing PAGE gel. For a 30 mL gel, add; 18 Ω H_2_O (22 mL), Acrylamide/Bis 29:1% (9 mL) and 5× TBE (3.5 mL), to a 50 mL tube. Close the lid tightly and gently invert the solution several times. Under the fume hood, loosen the lid and allow the mix to de-gas for 15 min. To cast the gel, add; 400 μL of 10% APS, and 40 μL of TEMED. Close the tube and gently invert the solution several times. Cast the gel and allow polymerizing for a minimum of 2 h.
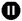
**PAUSE STEP** PAGE gels can be stored for a few days at 4 °C–after the gel solidifies, dampen lab tissue with 1× TBE buffer and cover the top and bottom of the gel plates, seal the gel in a plastic bag. Do not store PAGE gels for more than a few days.
4. Prepare the PAGE gel in 1× TBE running buffer and load 7.5 μL of the samples from the temperature ramp-down (N.B. Phusion Green Master Mix contains loading dye). Load an appropriate DNA ladder, negative PCR control, and a positive control (wildtype only PCR amplicon). Run the gel at 50 V for 1 h, then increase to 200 V for 2.5 h. After the run, remove the gel from the plates, wash it three times with distilled water, submerge the gel in 75–100 mL of distilled water and add 5 μL of ethidium bromide (10 mg/mL), gently shake for 15 min. Image the gel on a Gel Doc™ XR+.

Note 1: With four or more egg batches, it will be more efficient to first screen pooled G_1_ egg batches, by combining five to ten eggs in one microtube, and following steps 3, 4, 6, 7 and Section 3.1.2. Then select the specific egg batches bearing DNA modifications and follow this protocol from step 3, using single eggs as samples.

Note 2: As an alternative to the heteroduplex assay direct sequencing of the PCR products and analysis by using specific software [e.g., Tracking of Indel by Decomposition (TIDE https://tide.nki.nl/ [25]) or ICE Analysis (https://ice.synthego.com/) [26]] is possible. These analyses provide computational readouts allowing the identification of egg batches carrying mutant G_1_ individuals. Carefully evaluate which approach to take depending on the number of samples, as direct sequencing and computational analysis are less labor-intensive but more time and cost demanding compared to heteroduplex assay.

### 3.2. Mutant Sequence Determination

PCR, Cloning and Sanger sequencing. Time for Completion: PCR–3 h; TA cloning 3 h; transformation 2 h with overnight (ON) culture; colony selection 1 h with ON culture; plasmid miniprep 90 min. per 24 samples.

1. Perform PCR using standard Taq polymerase and the gDNA previously extracted from the known mutated G_1_ egg samples (see point 2 in Section 3.1.1). Prepare 480 μL of DreamTaq master mix as in Table 3 (see primer sequences in Figure 1 and Table 1). Vortex briefly and centrifuge.
2. Add 19 μL of the master mix to a PCR tube for each sample and add 1 μL of G_1_ embryonic gDNA (20 ng/μL), prepare a positive control with wildtype gDNA (20 ng) and a negative control with 1 μL of H_2_O. Briefly vortex and centrifuge the mixtures. Perform PCR (95 °C for 3 min, [95 °C for 30 s–51 °C for 30 s–72 °C for 45 s] × 25, 72 °C 10 min.) and store the PCR products at 4 °C. Run 5 μL of each on a 1.2% agarose gel (as in point 6 in Section 3.1.1) to confirm all samples have amplified the fragment.
3. Dilute the PCR products 1:10 in NF H_2_O. Ligate the PCR amplicons into the StrataClone PCR Cloning vector, following the manufacturer’s protocol. Perform one ligation reaction for each sample.
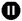
**PAUSE STEP** Ligated plasmids can be stored as a reaction mixture at −20 °C before transformation.
5. Transform competent cells following the provided protocol and incubate ON at 37 °C.
6. Select at least five colonies from each ON plate. Aseptically prepare five 5 mL-aliquots of sterile LB broth with ampicillin (100 μg/mL) in 15 mL tubes. Using a sterile pipette tip inoculate each 5 mL LB aliquot with one colony. Incubate at 37 °C with vigorous shaking ON.
7. Isolate the plasmids using the PureYield™ Plasmid Miniprep System (Promega, Dane County, WI, USA) following the manufacture’s protocol. Elute the DNA in NF H_2_O (required for Sanger sequencing). Use a NanoDrop to check the quality and quantity of the samples.
8. Prepare the plasmid DNA for Sanger sequencing following the service provider’s guidelines.
9. A protocol for computational analysis of Sanger sequences is available in Supplementary Methods A.
10. Design ARMS-PCR primers for the appropriate mutation/s identified by Sanger sequencing (Table 1).
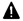
**CRITICAL STEP** Test the ARMS-PCR primers using wildtype gDNA. Mutant (M)-ARMS-PCR primers must not generate an amplicon using wildtype gDNA.

### 3.3. Hemolymph Sampling and Screening with ARMS-PCR

#### 3.3.1. Hemolymph Sampling and Larvae Rearing. Time for Completion: Hemolymph Sampling-2–3 h Per 50 Worms. DNA Extraction–90 min. Per 24 Samples. Larvae Rearing–6–7 Days for the 5th Instar and 12–14 Days for Pupation and Adult Emergence

**Figure 6:**
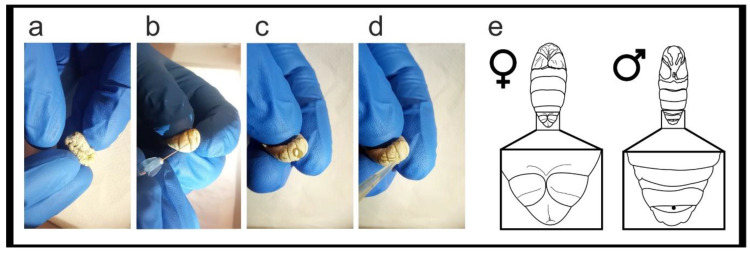
Hemolymph sampling and sexing pupae. To sample larval hemolymph, (**a**) fold the larva, stretch and expose its dorsal surface; (**b**) gently pierce between the dorsal folds of the dermis with a hypodermic needle (**c**) a drop of hemolymph forms at the wound site; (**d**) collect the hemolymph with a micropipette; (**e**) morphological characteristics used to sex pupae, females bear a vertical line on the penultimate (8th) segment, males bear a whole penultimate segment and have a spot on the 9th segment.

1. Rear G_1_ egg batches bearing the mutation until the 1st–2nd day of the 5th larval instar.
2. For each positive G1 batch, process at least 30–50 larvae.
3. Sterilize the workspace with 70% ethanol. For each larva, label one 1.5 mL microtube and one sterile Petri dish with the same code. Repeat for each larva, in advance. Place the labeled microtubes on ice, on the workbench near the labeled Petri dishes.
4. On the workbench, prepare; a small beaker with 50 mL of 70% ethanol, lab tissue, a microtube rack, a P20 micropipette, sterile tips, hypodermic needles, and waste containers for general lab waste, and hypodermic needles.
5. Using forceps, select a larva and carefully submerge it into the 70% ethanol beaker for 3 s. Gently blot the larva dry on lab tissue and let air dry for a few seconds. While air drying, open a labeled microtube and take its paired Petri dish.
6. Pick up and gently fold the worm head-to-tail, exposing the dermis between the body folds (Figure 6a). Hold the larva over the microtube and use a hypodermic needle to gently pierce the dermis without deeply entering the body cavity (Figure 6b), a drop of hemolymph will pool at the wound (Figure 6c).
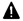
**CRITICAL STEP** Collect the hemolymph drop using a P20 micropipette (Figure 6d) and place into the microtube. Do not squeeze the larvae or attempt to withdraw additional hemolymph from the wound with the micropipette. Store the hemolymph on ice.
8. Place the larva into the appropriately labeled Petri dish. Continue sampling remaining larvae. Allow the sampled larvae heal for a minimum of 20 min. After healing, feed larvae in the Petri dishes.
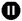
**PAUSE STEP** Hemolymph samples can be frozen at −20 °C until use.
10. To rear sampled G_1_ larvae: everyday, remove the Petri dish lid and place upside down. Using forceps, transfer the larva to the lid, empty the frass and dry food from the dish, if necessary wipe the dish with ethanol soaked lab tissue before transferring the larva back with fresh food.
11. After 6–7 days the larvae will prepare to spin their cocoon. Empty and clean the dish, add a folded square of lab tissue to facilitate cocoon spinning. After spinning begins do not interfere with the larvae for 4 days, then the pupae can be cut from the cocoons and sexed (Figure 6e).

#### 3.3.2. DNA Extraction from Hemolymph and Screening with ARMS-PCR

1. Perform gDNA extraction on 20 μL of hemolymph per sample using the PureYield Plasmid Miniprep System, following the manufacturer’s protocol. Using a NanoDrop, determine the samples quality and quantity. Prepare 20 ng/μL working gDNA solutions for each sample.
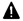
**CRITICAL STEP** High fidelity polymerase cannot be used for ARMS-PCR. Prepare PCR master mixes for both wildtype (WT) and mutant (M) ARMS-PCR reactions as in Table 4.
3. For each gDNA sample, prepare two PCR tubes with 9 μL of the WT-ARMS –PCR and M-ARMS-PCR master mixes. Add 1 μL (20 ng) of gDNA to both reactions. Repeat for each individual gDNA sample. Include a negative control, a positive control is not required as each reaction contains internal positive control primers. Vortex briefly and centrifuge the mixtures. Run the PCR (95 °C for 3 min, [95 °C for 30 s–51 °C for 30 s–72 °C for 45 s] × 34, 72 °C for 10 min.) and store the PCR products at 4 °C or use immediately.
4. Prepare a 1.2% agarose gel with 1 × TAE buffer and stain with ethidium bromide. Load a DNA ladder (5 μL of GeneRuler 1 kb) into the first well. Always be consistent with sample loading: load 5 μL of the WT-ARMS-PCR reaction followed by 5 μL of the M-ARMS-PCR reaction for an individual hemolymph gDNA sample in order to easily compare the two reactions. Run the gel (30 min at 100 V) and image on a GelDoc XR+.
5. Maintain G_1_ larvae showing a positive amplification M-ARMS reaction and discard those showing only a positive amplification WT-ARMS reaction. Rear larvae until the adult stage and in-breed G_1_ moths bearing mutation to generate the G_2_ egg batches. Notes for breeding *B. mori* moths can be found in Supplementary Methods B.
6. Rear G_2_ larvae until the 5th instar and perform DNA extraction from hemolymph and ARMS-PCR as from Section 3.3.1.
7. Select hemi- and homozygous mutant G_2_ larvae and rear until adult stage. Mate them to generate a G_3_ mutant stable line.

## 4. Expected Results

### 4.1. Initial Identification of Mutations

**Figure 7:**
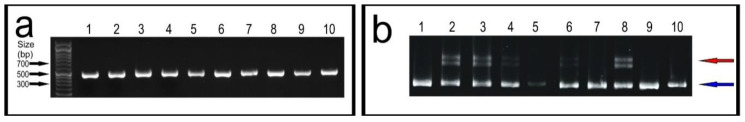
Screening for DNA modifications. (**a**) *per* amplicons from individual embryos in a 1.2% agarose gel. (**b**) 15% PAGE gel heteroduplex assay of samples reported in (**a**). Blue arrow indicates the homoduplexes and red arrow the heteroduplexes.

Successful PCR of the target region should show a clear single band in a 1.2% agarose gel (Figure 7a). Following heteroduplex formation by temperature ramp-down, the PCR products are analyzed by PAGE. G_1_ eggs carrying a mutation can be identified from the interpretation of the heteroduplex assay results. In fact, wildtype samples and the positive control will show a single band, corresponding to a homoduplex fragment (Figure 7b). Samples carrying a mutation will appear with larger multiband, corresponding to heteroduplexes (Figure 7b). DNA mutant samples are then selected for subsequent cloning and Sanger sequencing.

### 4.2. Mutant Sequence Determination

**Figure 8:**
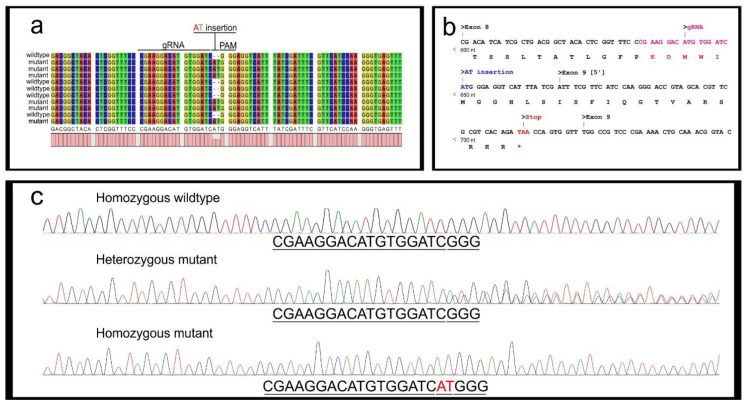
Mutant sequence determination. (**a**) *per* amplicon Sanger sequencing *of* five clones derived from two heterozygous egg samples, aligned with a wildtype *per* sequence. An AT insertion upstream of the PAM site is identified in several sequences; (**b**) *per* DNA and protein sequences. The AT insertion at the DNA level causes a frameshift mutation resulting in a truncated PER protein composed of 220 amino acids (full length *per* = 1108 aa). (**c**) Explicative chromatograms of: homozygous wildtype (**top**) and mutant (**bottom**) embryos generate clear single peaks, a heterozygous individual (**middle**) generates double peaks downstream of the PAM site.

Following cloning, Sanger sequencing will return either wildtype or mutated sequences (Figure 8a). The identified mutation is then analyzed to observe whether a stop codon is generated in the protein-coding sequence (Figure 8b).

As a note, PCR samples derived from G_1_ embryos could also be processed with direct Sanger sequencing. Homozygous wildtypes will generate clear single peaks throughout the chromatogram, while heterozygous mutant samples will show double peaks downstream of the PAM site (Figure 8c). It is important to mention that genes targeted on the Z sex chromosome will result in hemizygous females either positive or negative for the mutation. Samples from hemizygous females appear identical to homozygous samples (either wildtype or mutant).

### 4.3. Screening with ARMS-PCR

**Figure 9:**
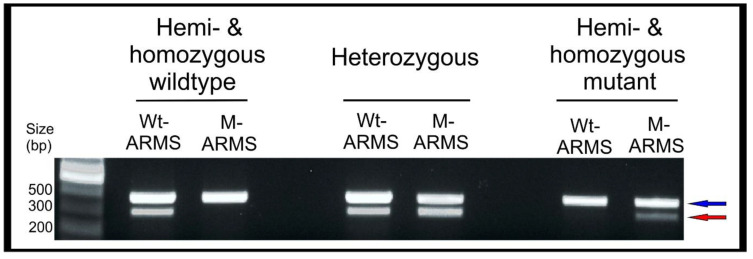
ARMS-PCR screening showing the banding patterns for hemi- and homozygous wildtype (**left**), a heterozygous (**middle**) and hemi- and homozygous mutant (**right**) samples. Arrows indicate the internal cyc control (**blue**) and the per amplicon (**red**).

The mutant and wildtype sequences are used to design ARMS-PCR primers. We designed two forward primers, ARMS_WT_FOR and ARMS_M_FOR respectively able to amplify the wildtype or mutant sequences, when used with the same reverse *pe*r_REV primer (Table 1). The expected banding patterns for successful ARMS-PCR are shown in Figure 9. All samples will show a band corresponding to the internal PCR control (*cyc* amplicon). A *per* wildtype sample will produce a *per* amplification band in the WT-ARMS reaction and no *per* amplification bands in the M-ARMS reaction. A heterozygous larva will produce *per* amplification bands in both reactions. In contrast, a homozygous mutant will produce no *per* amplification bands in the WT-ARMS reaction and a *per* amplification band in the M-ARMS reaction. As *per* is located on the Z sex chromosome, females will be hemizygous wildtype or mutants, only displaying a single band in either the wildtype or mutant ARMS-PCR, respectively.

To establish a stable *per* mutant line, only G_1_ females bearing a *per* amplicon in the M-ARMS-PCR reaction and G_1_ males bearing a *per* amplicon in both ARMS-PCR reactions should be selected, followed until adult stage, and crossed to generate G_2_ progenies. G_2_ 5th instar larvae will be screened via hemolymph DNA extraction and ARMS-PCR, identifying the appropriate hemizygous and homozygous mutant individuals to -found the stable mutant line, from G_3_ onward.

## 5. Conclusions

CRISPR/Cas9 is an efficient and relatively simple gene-editing technology rapidly becoming the technique of choice for the generation of novel gene knock-out and knock-in research organisms. A variety of screening methods are available to determine the nature of CRISPR/Cas9 induced mutations and mutant baring individuals, and screening workflows should be tailored to specific target species. Here, we have reported an efficient screening protocol to identify CRISPR/Cas9-induced indels in a Z-linked gene in the silkworm *Bombyx mori* and have applied to generate a stable mutant line. This workflow can be employed with standard molecular biology equipment and can be generally used to identify a wide range of CRISPR/Cas9-mediated mutations within genes localized on both autosomes and heteromorphic sex chromosomes. This method could also be extended to detect CRISPR/Cas9-mediated mutations in other model and non-model insects, including haploid insects.

## Figures and Tables

**Table 1:** PCR primers for standard and ARMS-PCR.

Primer Name	Gene	Accession no.	Start Position	End Position	Fragment Length (bps)	Sequence (5′–3′)
per_FOR	*period*	LOC692522	10,240	10,259	482	ACTGCATGACGGCAACTTT
per_REV			10,702	10,722		TGCCGTTCCTTTTGAAATTC
ARMS_WT_FOR	*period*	LOC692522	10,417	10,435	305	GAAGGACATGTGGATCGG
ARMS_M_FOR			10,417	10,437	307	CCGAAGGACATGTGGATCAT
cyc_FOR	*cycle*	LOC692530	69,748	69,774	541	CCTGAATAGTTACCAAATACATTTGA
cyc_REV			70,265	70,286		CGAATTTTGGTGGTCGTGTAT

In bold the 3′ nucleotides of the ARMS-PCR primers for detecting the wildtype and mutated in the per target region.

**Table 2:** Setup of Phusion PCR master mix.

Reagent	Concentration	Volume (μL)
Phusion Green Master Mix	2×	250
per_FOR	10 μM	25
per_REV	10 μM	25
H_2_O	-	180

**Table 3:** Setup of DreamTaq master mix.

Reagent	Concentration	Volume (μL)
DreamTaq (2×) mix	2×	250
*per*_FOR	10 μM	25
*per*_REV	10 μM	25
H_2_O	-	180

**Table 4:** Setup of two PCR master mixes for ARMS-PCR screening.

Reagent	Concentration	WT-ARMS-PCR vol. (μL)	M-ARMS-PCR vol. (μL)
DreamTaq Green	2×	250	250
*cyc*_FOR	10 μM	25	25
*cyc*_REV	10 μM	25	25
*per*_REV	10 μM	25	25
ARMS_WT_FOR	10 μM	25	0
ARMS_M_FOR	10 μM	0	25
H_2_O	-	150	150

## Supplementary Materials

The following are available online at https://www.mdpi.com/2409-9279/4/1/4/s1, Supplementary Methods A-Sequence alignments; Supplementary Methods B-Notes on breeding *B. mori.*
