# Supplementary material for: An Efficient Workflow for Screening and Stabilizing CRISPR/Cas9-Mediated Mutant Lines in Bombyx mori
Source: Methods Protoc. 2020 Dec 29;4(1):4. doi: 10.3390/mps4010004 (PMC7839013; doi:10.3390/mps4010004)
Supplement: Supplementary file 1 [file mps-04-00004-s001.pdf]

*methods and protocols*

# An Efficient Workflow for Screening and Stabilizing CRISPR/Cas9-Mediated Mutant Lines in *Bombyx mori*

**Daniel Brady [1,*], Alessio Saviane [2], Silvia Cappellozza [2] and Federica Sandrelli [1,*]**

[1] Department of Biology, University of Padova, via U. Bassi 58/B, 35131 Padova, Italy
[2] Council for Agricultural Research and Economics, Research Centre for Agriculture and Environment, Sericulture Laboratory, 35143 Padova, Italy; alessio.saviane@crea.gov.it (A.S.); silvia.cappellozza@crea.gov.it (S.C.)
* Correspondence: daniel.brady@unipd.it (D.B.); federica.sandrelli@unipd.it (F.S.); Tel.: +39-049-8276216

## Supplementary Methods

**Supplementary Methods A: Sequence Alignments**

Computational analyses to determine the specific DNA modifications and subsequent amino acid modifications can be completed with a large number of software packages. Here, we describe the process using three freely available software packages, namely: CLC Sequence Viewer (V.8, Qiagen), MEGA 7, and Serial Cloner.

1. Download the sequence files and rename them with their appropriate code and replicate numbers, if required. Import the files into the CLC Sequence Viewer. Drag and organize the sequences into a logical order, starting with the wildtype, then egg batch_replicate.
2. Perform a sequence alignment (Toolbox –> Alignments and Trees –> Create Alignment); in the dialogue box, select the trimmed sequences, including the wildtype sequence; multiple items can be selected by holding the "Ctrl" button. Click the blue right arrow to move the items to the "Selected Elements" window. Select "Next", in set parameters select "Next", and in the result handling dialogue box, select "Finish".
3. If plasmid specific primers were used for sequencing, first trim the plasmid regions from the aligned sequences, then paste the forward PCR primer sequence into the search box (Ctrl + F). Highlight and delete the upstream regions (right-click–delete sequence). Repeat this process with the reverse PCR primer, but deleting the 3′ downstream data.
4. N.B., using a blunt-ended cloning vector (PCR with high-fidelity polymerase) will result in inverted fragments. Therefore, the sequences should be trimmed before an initial sequence alignment (perform Step 3 before Step 2). Note the samples not aligned to the wildtype fragments and reverse complement these sequences (Toolbox -> Nucleotide Analysis -> Reverse Complement Sequence); in the dialogue box, select all of the required samples and click the blue "right" arrow. Select "Next", and then select "Finish". Save the files and perform a new sequence alignment as in Step 2.
5. Appropriately rename and save the sequence alignment. Copy and paste the gDNA region of gRNA into the search box. The search function will highlight the wildtype target site, and any mutations in the egg batches will be visible. Note the samples containing mutations.
6. In MEGA 7, open the wildtype chromatogram and samples containing a mutation; check that the sequences have clear individual peaks for the base reads. Use the "copy and paste" function with around 20 nucleotides downstream of the gRNAs PAM site to confirm the mutation is present; CRISPR induced mutations usually occur upstream of the PAM.
7. Checking for stop induced codons: Open SerialCloner (2.6.1), click "File" then "New" and paste the wildtype transcript into the dialogue box. Name and save the file as the wildtype.
8. In the file directory, copy the wildtype file and rename it with the appropriate Sanger sequencing code. Repeat for every unique mutation identified by the Sanger sequencing.
9. Open the first mutated sequence file, copy and paste the genomic region of the gRNA into the search function (Ctrl + F), and edit the genome to contain the exact mutation identified by the Sanger sequencing. Save the file. Highlight the mutated sequence, right-click and select "create new feature from selection", label the mutation appropriately, and change the color. Save the file. In the dialogue box, click on "Sequence Map" and select the correct translation reading frame. The sequence region will appear annotated (and colored) as the feature previously created. Search downstream for the presence of stop codons (indicated by *). Repeat for each unique mutation. Note: In the correct reading frame, indels in multiples of 3 bps will not introduce stop codons.

10. Export high quality sequence maps: Select "Print" and a Page Setup dialogue box will appear, select "OK", change the selected printer to "Print in PDF" and title the PDF document and select a file location.

**Supplementary Methods B: Notes on *B. mori* Breeding**

- Perform mating, egg-laying, and development at 25 ± 1 °C; embryo development should be carried out with 85 ± 5% relative humidity.
- For the initial outbreeding of $G_0$ injected individuals, separate the rear wildtype silkworm with the same genetic backgrounds, in parallel. Mate $G_0$ injected moths with wildtype animals with the same genetic background, but not with other $G_0$ injected individuals, even if they are shown to be wildtype through screening. This will help minimize the retention of off-target edits in the final homozygous line. Possible CRISPR/Cas9 off-target effects can also be avoided/limited with additional outbreeding of subsequent generations ($G_1$, $G_2$, etc.).
- Adult eclosion in *B. mori* is strongly driven by light, with most adults beginning to emerge after light-on, spanning over several days. However, some eclosion can occur throughout the day. Therefore, with a limited number of animals for in-breeding, attention should be paid to the pupae throughout the day.
- Adult *B. mori* will mate immediately upon eclosion. If a suitable pair emerge, place the male and female together on a sheet of paper. The male will immediately begin vibrating his wings while searching for the female before mating. Cover the pair with a paper cup and allow them to copulate for 2 h. Remove the male and place a paper cup over the female, who should be laying eggs by the following morning.
- One male can be mated with up to three females if required.
- If only a few females are present, breed them with two or three males for 2 h each.
- Smaller males may find it difficult to mate with a larger female. If the pair cannot be coupled within 1 h, try to pair them with other moths.
- Female moths will lay eggs regardless of whether or not they copulate. If no suitable male is available, i.e., there are more females than males, then delay egg-laying. Moths can be stored at 5 °C for two to three days until a suitable mate emerges, although prolonged exposure at 5 °C is increasingly deleterious.
- Pool egg batches with an identical mutation.
